# Public health relevance of drug–nutrition interactions

**DOI:** 10.1007/s00394-017-1510-3

**Published:** 2017-07-26

**Authors:** Szabolcs Péter, Gerjan Navis, Martin H. de Borst, Clemens von Schacky, Anne Claire B. van Orten-Luiten, Alexandra Zhernakova, Renger F. Witkamp, André Janse, Peter Weber, Stephan J. L. Bakker, Manfred Eggersdorfer

**Affiliations:** 1 DSM Nutritional Products Ltd., Wurmisweg 576, 4303 Kaiseraugst, Switzerland; 2 University of Groningen, University Medical Center Groningen, Hanzeplein 1, 9713 GZ Groningen, The Netherlands; 3 Preventive Cardiology, Medizinische Klinik und Poliklinik I, Ludwig Maximilians-Universität München, Ziemssenstr. 15, 80336 Munich, Germany; 4 Omegametrix GmbH, Am Klopferspitz 19, 82152 Martinsried, Germany; 5 Division of Human Nutrition, Wageningen University, Stippeneng 4, 6708 WE Wageningen, The Netherlands; 6 Department of Geriatric Medicine, Hospital Gelderse Vallei, Willy Brandtlaan 10, 6716 RP Ede, The Netherlands; 7 University of Hohenheim, Schloß Hohenheim 1, 70599 Stuttgart, Germany

**Keywords:** Public health, Drug–nutrient interactions, Micronutrient deficiency, Microbiota, Health benefits

## Abstract

The public health relevance of drug–nutrition interactions is currently highly undervalued and overlooked. This is particularly the case for elderly persons where multi-morbidity and consequently polypharmacy is very common. Vitamins and other micronutrients have central functions in metabolism, and their interactions with drugs may result in clinically relevant physiological impairments but possibly also in positive effects. On 12 April 2016, the University Medical Center Groningen (The Netherlands), as part of its Healthy Ageing program, organized a workshop on the public health relevance of drug–nutrient interactions. In this meeting, experts in the field presented results from recent studies on interactions between pharmaceuticals and nutrients, and discussed the role of nutrition for elderly, focusing on those persons receiving pharmaceutical treatment. This paper summarizes the proceedings of the symposium and provides an outlook for future research needs and public health measures. Since food, pharma and health are closely interconnected domains, awareness is needed in the medical community about the potential relevance of drug–nutrition interactions. Experts and stakeholders should advocate for the integration of drug–nutrition evaluations in the drug development process. Strategies for the individual patients should be developed, by installing drug review protocols, screening for malnutrition and integrating this topic into the general medical advice.

## Introduction

The University Medical Center Groningen (UMCG, The Netherlands) organizes yearly symposia on different, actual topics in the overlapping fields of medicine and nutrition as part of its Healthy Ageing program. Over the past number of years this event evolved to a think-tank, resulting in several publications, educational- and research collaborations [1–3]. The aim of the 2016 meeting that took place on 12 April at the UMCG was to evaluate the role and impact of drug–nutrition interactions for people using medication, especially the elderly. The focus was on current drugs, however, with the ambition to also provide input for future drug development by engaging experts from both academy and industry. The overall goal of the meeting was to create awareness, to provide an overview of this topic and to foster collaborative activities. Drug–nutrient interactions are often preventable or curable, provided that they are recognized in an early stage. Therefore, more insight in this matter will contribute to an improved health and well-being of patients. This paper summarizes the main messages and conclusions of the presentations and following discussions and advocates to take actions and integrate the topic “drug–nutrition interactions” in scientific research programs and in clinical practice.

## Clinical assessment of interactions of drugs with nutrients: an urgent unmet need

Whereas drug–drug interactions are widely recognized as clinically relevant and are included in most pharmacovigilance systems, nutrient–drug interactions are underexplored, and their assessment is not part of the clinical routine. Yet, there is ample data supporting the presence and relevance of nutrient–drug interactions, indicating that a systematic assessment would be necessary.

The poor translation of results from randomized clinical pharmacological trials to real life is one of the major challenges in medicine of the twenty-first century, as noted in the *Lancet* series: “Increasing value, reducing waste” [4]. Variation in—non-documented—environmental interacting factors, such as nutrients, or adverse drug effects on nutrient status, may be among the contributing factors. This becomes an even more urgent issue in the era of ageing societies, with a steady increase in the number of elderly with multimorbidity and hence increasing prevalence of polypharmacy [5] (Table 1). Awareness should be created that multimorbidity is no longer a confounder but the focus in this age group [6]. In general, pharmacotherapy is approached as the sum of separate medicinal treatments where multi-level interactions are not tested, and as such is poorly suited to the needs of the ageing individual. High costs, several adverse effects, conflicting guidelines are involved and this complexity makes it prone to multiple errors. Examples of specific interactions have been recently summarized and discussed elsewhere [7].

**Table 1:** Effects of polypharmacy on nutritional status

Effect on	Effect by
Food intake	Gastrointestinal discomfort/poor appetite
	Gastrointestinal digestion/absorption
	Central nervous system depression
Nutrient absorption	Gastrointestinal malabsorption
Metabolism and elimination of nutrients	Organ impairment (liver, kidney)
	Loss of nutrients essential to metabolism

## Drug–nutrition interactions as adverse drug reaction

### Adverse drug reactions (ADRs)

**Fig. 1:**
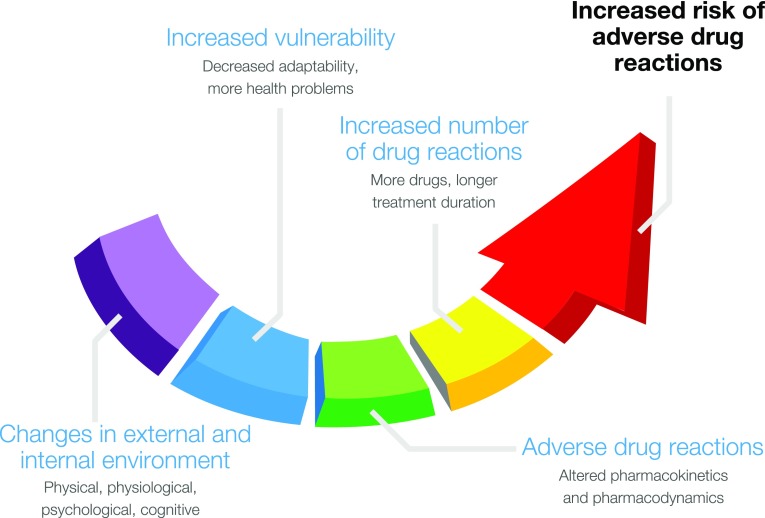
Increased risk of adverse drug reactions in older people

Medication-related health problems are a public health issue of major relevance, especially in elderly people. In the Western world, unintended, negative effects of drug use would be cause number three of mortality, immediately after cardiovascular disease and cancer. In the European Union, drug side effects are estimated to be responsible for 197,000 deaths per year [8]. The risk of adverse drug reactions (ADRs) increases with advancing years [9]. This can be explained by age-related changes in pharmacokinetics and pharmacodynamics, the increased vulnerability of elderly people, and by the high prevalence of multi-morbidity and consequently polypharmacy in this age group [10], as the number of medications used is the most important predictor of the risk of ADRs [7, 9] (Fig. 1). Median prevalence of adverse events for elderly in ambulatory care is reported to be 23% (IQR 19–31%) [11]. Prevalence of ADR-related hospital admissions of 75+ years subjects are estimated to be 10% (IQR 8–13%), of which 40% could have been prevented [12]. These figures may be underestimations, as 94% of the potential ADRs are suspected not be reported by health care practitioners, according to a systematic review of drug surveillance data from 12 countries [13]. Also in the premarketing phase, risk assessment has its limitations: drug development lines may be too short for long-term effects to become clinically manifest and the number of patients during the application phases is generally insufficient to adequately detect rare events [14]. Moreover, children, premenopausal women, and, elderly subjects are underrepresented in premarketing trials, just as patients having more than one disease and/or using more medications simultaneously.

### Drug–nutrition interactions (DNIs)

However, evaluation of drug safety before and after drug approval is principally about the consequences of single drugs or interactions between drugs. With exception of the effects of whole meals and macronutrients on drug action, drug–nutrition interactions (DNIs) are not specifically addressed during the drug development phase [15]. Recognition of DNIs is often hampered by limited nutritional knowledge among researchers and professionals in the health care field: Nutrition education in EU medical schools is between 0.5–3 days, and in the US about 2.5 day [16–18]. The lack of nutritional expertise is a plausible contributor to the already substantial underreporting of ADRs. Although drug and supplement use is increasing [19], and in particular in elderly populations, prevalence of polypharmacy and malnourishment is high [20], we can only speculate about the clinical relevance and public health impact of interactions between drugs and nutritional determinants. Theoretically, however, the impact on public health might be substantial, considering the clinical relevance of minimal adverse drug effects and an adequate nutritional status for health and well-being.

### Tool to structure DNIs

A helpful tool to address the relatively unexplored domain of DNIs is a classification framework for categorizing DNIs. A first distinction is made between the effects of meals, individual foods, specific nutrients or nutrition status on drug action, and vice versa, the effects of drug use on determinants of nutrition status [15, 21]:

- Class 1—effect of obesity and malnutrition on drug action,
- Class 2—effect of nutrition on drug action,
- Class 3—effect of specific nutrients or dietary supplements on drug action,
- Class 4—effects of drugs on nutrition status,
- Class 5—effects of drugs on nutrient status.

Class 1 DNIs are about the impact of overweight or malnourishment on drug action. A Class 2 DNI is illustrated by dietary fat and lapatinib, a protein kinase inhibitor for the treatment of metastatic breast cancer. Compared to fasting, the bioavailability of this antineoplastic agent increases by 168% after a low fat meal and by 325% after a high fat meal [22]. Bioavailability of abiraterone, a CYP17 inhibitor for the treatment of metastatic prostate cancer, even increases tenfold after a high fat meal [23]. These Class 2 interactions may ultimately be related to considerable cost savings in cancer treatment [22], as the drug leaflets of these oncology drugs indicate that the medication should be taken without any food. Class 3 DNIs initiated by the concurrent use of drugs and supplements may have advantages as well as risks [24]. Beneficial are the increased plasma levels of chemoresistance-inducing fatty acid 16:4(n-3) observed in human volunteers after consumption of fish and fish oil [25]. Oppositely, calcium chelating with a tetracycline medication in the gastrointestinal tract is a well-known example of an undesirable Class 3 interaction. The Dutch pharmacovigilance Centre Lareb, however, received only 55 reports of these adverse food–drug interactions over 25 years (1991–2014), which makes it likely that Class 3 DNIs are underreported [26]. Class 4 DNIs refer to the effects of drugs on nutrition status on macrolevel, which are influenced by a diversity of determinants: from the ability to see, smell, taste, eat, and drink, until the final excretion of urine and feces. Several publications report inverse associations of polypharmacy and use of specific drug groups with risk of malnourishment [27, 28]. The last category, Class 5 DNIs are about the links between drug use and nutrient status. In a sub-cohort of 631 geriatric outpatients of the Dutch PanDeMics study, for example serum 25-hydroxyvitamin D (25(OH)D) was 10 mmol/l lower (*p* = 0.01) in male subjects using ≥10 medications compared to males using fewer medications. Severe polypharmacy was present in 21% of the male patients. Also use of oral antidiabetics, vitamin K antagonists, diuretics, and ACE inhibitors was found to be significantly associated with lower vitamin D levels [29]. Depending on the cut-off value, prevalence of vitamin D deficiency in this cohort was 50% (25(OH)D <50 nmol/l) or 76% (25(OH)D <75 nmol/l). Other drugs, clearly affecting circulating vitamin D are anti-epileptics, anti-oestrogens, anti-virals, glucocorticosteroids and bisphosphonates [30]. By taking the pharmacological mechanism and localization of the interaction into account, the individual classes can be further split up into subclasses [15, 21].

## Interaction between oral contraceptives and folate

Neural tube defects (NTDs) are a group of congenital birth defects that affect the central nervous system; the most common types of NTD are spina bifida and anencephaly. Neural tube closure takes place between 24 and 28 days after conception, thus NTD’s occur very early in pregnancy, even before most women know that they are pregnant. Each year, ca. 300,000 babies are born with NTD globally, while in Europe ca. 4500 pregnancies are affected. Regional prevalence varies considerably due to differences in environmental (e.g., dietary) and genetic factors [31]. In a study, conducted in Dutch school-aged children whose mothers had low folate status during early pregnancy, cognitive development was assessed. Low prenatal folate levels were associated with smaller total brain volume, poorer language performance, reduction in memory and learning, and decrease in visio-spatial domains. These results indicated that insufficient folate status in early pregnancy had long-lasting effects on the offspring’s cognitive development [32]. A review article examined whether folate supplementation before and during early pregnancy could reduce NTD and other birth defects without causing adverse outcomes for mothers or babies. Authors concluded that folic acid, alone or in combination with other vitamins and minerals, prevented NTD and was safe for the mothers and newborns [33]. Furthermore, a dose–response study on red blood cell (RBC) folate during pregnancy was conducted: RBC folate levels of ≥906 nmol/L (=400 ng/mL) were identified as providing the greatest protection from NTD, however, insufficient data was available to calculate the risk reduction for levels ≥1292 nmol/L. It was also shown that a doubling of the RBC folate level is achievable with an increased folic acid intake of 0.4 mg per day. Importantly, it was also estimated that a 48% reduction in the total NTD rate was achievable with an increased population intake of 0.4 mg per day of folic acid [31, 34].

**Fig. 2:**
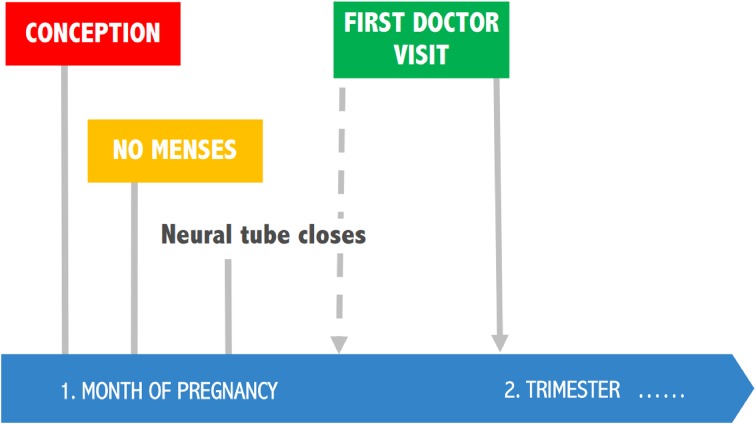
Folate status in women of reproductive age should be adequate to prevent neural tube defects (NTDs)

Surveys indicate that only a small proportion of women take folic acid supplements during the periconceptual period [31], and this is complicated by the issue that folate status is generally low in women. Current recommendations in the US and Europe propose that all women planning or capable of pregnancy should take folic acid supplements at doses of 0.4 mg/day, commencing at least one month before conception. However, implementation of these recommendations is unsatisfying: Only 24% of women of childbearing potential consume the recommended intake of folic acid in the US. After the introduction of public folic acid awareness campaigns in the UK, The Netherlands, and Australia, post-campaign rates of folic acid supplement use were below 50% of women of childbearing potential. In addition, there is evidence that use of oral contraceptives (OC) may reduce folate status. A systematic review and meta-analysis of the effect of oral contraceptive use on plasma and RBC folate concentrations showed that independently of OC use, almost all studies reported much lower mean RBC folate values than the protective cut-off levels. However, the mean difference in RBC folate concentrations between OC-users vs. non-users was clinically significant [35]. It is known that oral contraceptive use can lead to low serum and tissue concentrations of folate, but the mechanism of this effect is uncertain. According to some hypotheses, OC may interfere with the absorption of pteroylpolyglutamates or might cause malabsorption of dietary folate by inhibition of jejunal folate conjugase, however, this was not confirmed by in vitro studies. Oral contraceptive users have normal folate absorption but show increased serum clearance and urinary excretion of folates. A possible mechanism for increased folate excretion would be a change in serum binding proteins for folate. Low serum folate in OC users could also be explained by enzyme induction in the liver [36]. Estrogen levels of OC were consequently reduced from 150 µg in the 1960s to 20–50 µg after 1988, while many of the studies were conducted in the 1970s and evidence from trials using modern OC is very limited. Also, little information is available on confounders such as dietary folate. Wilson et al. states that insufficient data is available for a definitive conclusion regarding the effect of modern OC on folate status [37]. However, based on the above it is clearly confirmed and evident, that folate status in women of reproductive age should be adequate as neural tube closes before pregnancy (Fig. 2).

It has been proposed that folate-fortified OC are an efficient way to maintain adequate blood folate levels for women of reproductive age [31]. In an RCT, women received OC with folate or l-5-methyltetrahydrofolate (l-5-MTHF) for 24 weeks and OC alone for an additional 20 weeks (folate elimination phase). An OC fortified with folate or l-5-MTHF for 6 months lead to a significant increase and maintenance in folate status in women of reproductive age [38, 39]. Co-administration of folate with OC increased RBC folate levels above ‘NTD-protective’ threshold of >906 nmol/L within eight or ten weeks after commencement of regular use of l-5-MTHF or folate, respectively. The protective levels were maintained for 10 or 8 weeks after cessation of intake for l-5-MTHF or folate, respectively [40]. Thus, folate-fortified OC have the potential to protect against NTD, especially considering that about half of the pregnancies are unplanned, 21.1% of women become pregnant in one menstrual cycle after stopping OC use and 45.7% conceive within three cycles after stopping OC use.

## Interaction of vitamin D and vitamin K to prevent calcification

Despite the availability of lifestyle and pharmacological interventions targeting cardiovascular risk factors including hypertension, hypercholesterolemia, and diabetes, their efficacy to reduce cardiovascular risk is limited. This particularly applies to high-risk populations such as patients with chronic kidney disease [41]. The resulting high residual cardiovascular risk underscores the need to identify and target additional pathways. Vascular calcification contributes to stiffening of the vasculature, which is a main driver of cardiovascular diseases including heart failure with preserved ejection fraction (HFpEF). Vascular calcification is a complex process directed by the interplay between pro- and anti-calcification factors. Vitamin D and K both influence the process of vascular calcification. Although not a classical example of drug–nutrition interactions, the various preventive and therapeutic applications of both nutrients qualify the case for further elaboration within the context of this article.

Animal studies suggest that vitamin D plays a key role in the prevention of vascular calcification. Vitamin D receptor knockout mice display vascular calcification [42], and treating animals prone to develop calcifications with vitamin D provides vascular protection [43]. Importantly, exposing animals to excessive amounts of vitamin D (receptor activators) may have adverse effects, with enhanced vascular calcification [43]. This is a warning sign also observed in patients; a recent analysis of the PREVEND cohort showed that, although an inverse association between 25(OH) vitamin D and the risk of hypertension was observed, the opposite was the case for the active metabolite 1.25(OH)2 vitamin D [44]. Each 1-SD decrement of 1.25(OH)2 vitamin D was associated with a 10% lower hypertension risk, independent of potential confounders. The same dilemma was observed in the ViRTUE-CKD trial, which demonstrated that the vitamin D receptor activator paricalcitol lowers albuminuria in chronic kidney disease patients, but at the same time increases markers of deregulated phosphate homeostasis (i.e., fibroblast growth factor 23) and vascular calcification propensity [45]. Overall, it seems that (25-OH) vitamin D deficiency has the most relevant impact on clinical outcomes. A recent large meta-analysis showed that in the elderly, vitamin D deficiency is associated with a higher risk of cardiovascular mortality [46]. In the general population, the need for vitamin D supplementation to prevent mortality remains a matter of debate. In a Cochrane meta-analysis including data from 56 RCTs containing overall 95,286 participants, it was found that cholecalciferol (vitamin D3), but not ergocalciferol (vitamin D2), alfacalcidol or calcitriol reduced mortality upon long-term (mean 4.4 years) treatment [47]. The authors calculated that 150 people need to be treated with vitamin D3 over five years to prevent one additional death, which may seem reasonable from a public health perspective. In contrast, a recent trial sequential meta-analysis suggested a limited effect of vitamin D supplementation on non-skeletal outcomes [48]. However, the methodology used in this paper has been heavily criticized in the scientific community [49–51].

The vitamin K-dependent matrix-gla protein (MGP) is among the most powerful endogenous calcification inhibitors [52]. Several animal studies have shown that vitamin K insufficiency contributes to vascular calcification, which can be reversed by vitamin K supplementation [53–55]. In a state of vitamin K insufficiency, undercarboxylated MGP species including desphospho-undercarboxylated MGP (dp-ucMGP) are elevated in the circulation. Epidemiological studies have consistently shown independent associations of high circulating dp-ucMGP levels with both arterial stiffness [56] and adverse cardiovascular outcomes in high-risk patients including patients with aorta valve stenosis [57], cardiovascular disease [58], CKD [59], and renal transplant recipients [60]. Currently, ongoing clinical trials addresses the effect of vitamin K supplementation on vascular calcification in hemodialysis patients [61] and patients with coronary artery disease [62]. Higher vitamin K intake reduced dp-ucMGP [63], and has been associated with a lower risk of aorta calcification and (cardiovascular and all-cause) mortality in the general population. These observations suggest that increasing vitamin K intake could be a successful strategy to reduce cardiovascular risk [64].

Little is known about the interplay between vitamins D and K in relation to vascular calcification. The availability of a novel serum calcification propensity assay [65] will importantly facilitate studies prospectively addressing calcification risk. A higher calcification propensity has consistently been associated with a higher (cardiovascular) mortality risk in patients with CKD [66] and renal transplant recipients [67]. An integrated nutritional intervention, including the targeting of vitamin D and K deficiencies, may eventually turn out to be the most fruitful approach to retard vascular calcification and improve cardiovascular prognosis in high-risk patients.

## Omega-3 benefits

Previously, conventional research in nutrition has focused on dietary intake of the two marine omega-3 fatty acids eicosapentaenoic acid (EPA) and docosahexaenoic acid (DHA). However, this approach has inherent methodological issues: Food Frequency Questionnaires often result in implausible data, look up tables are usually outdated and issues of bioavailability, like inter-individual variability or dependency on matrix effects are ignored [68–71]. It is no wonder that there is no agreement of recommendations on this basis [72].

**Fig. 3:**
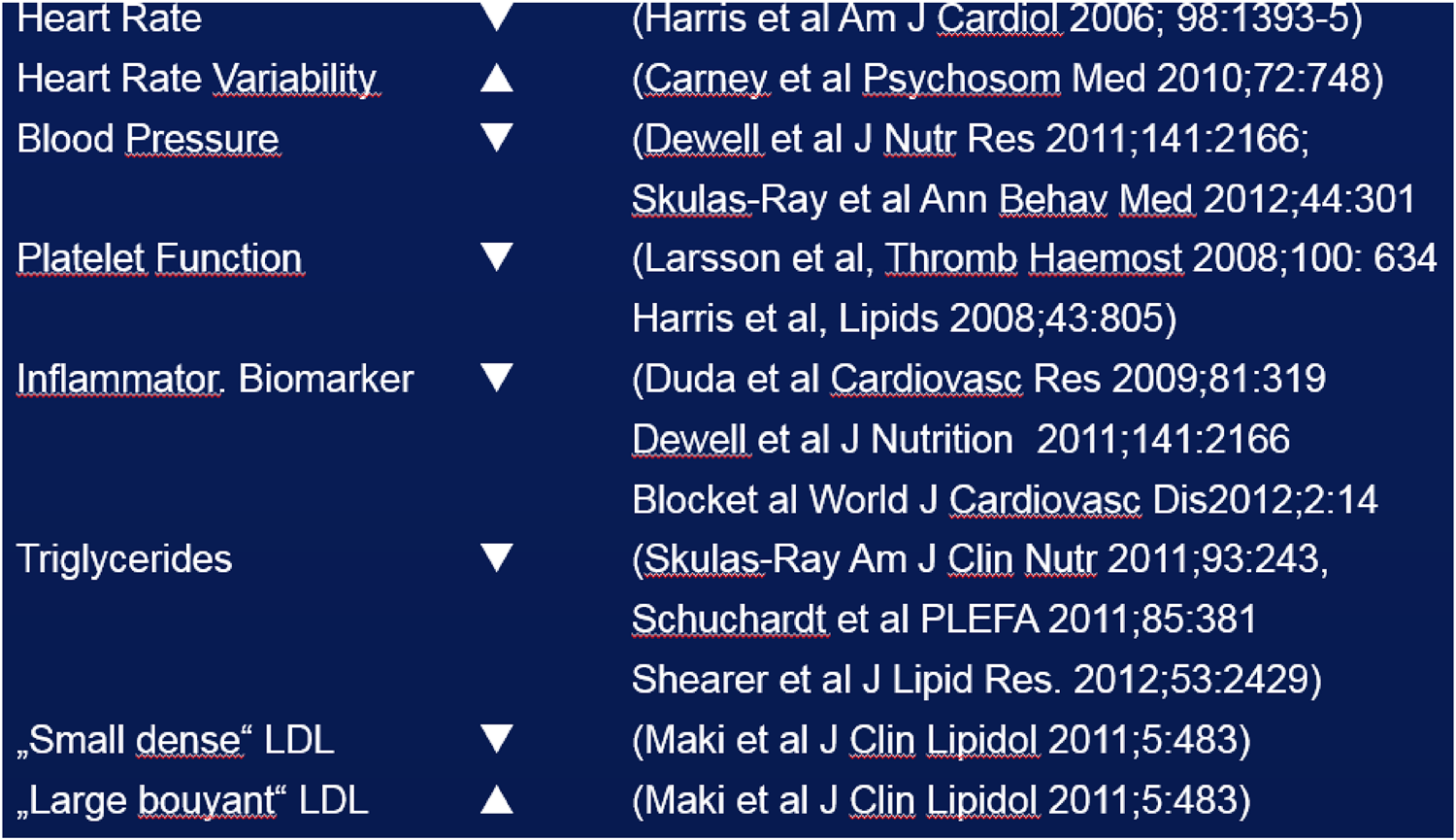
Effects of increasing the HS-Omega-3 Index on surrogate parameters

It is known that EPA and DHA content of many cells is reflected in erythrocytes, therefore, erythrocyte EPA plus DHA is used as a biomarker with low biological variability for the status of an individual in these two fatty acids. On this basis, a standardized analytical procedure, validated for 26 fatty acids, with low analytical variability has been established: The “HS-Omega-3 Index^®^” [73]. It is supported by the largest database of all methods of fatty acid analyses, backed up by 212 publications and >50 ongoing research projects. The HS-Omega-3 Index is representative for heart and breast tissues in humans, and in experimental animals it is representative for kidney, cerebral cortex, liver, lung and gut tissues; there is almost no correlation with intake [69, 74–77]. A target range for the HS-Omega-3 Index has been defined as 8–11% EPA plus DHA in total erythrocyte fatty acids [78]. Lower levels are associated with increasing risk for total mortality and cardiovascular events like fatal and non-fatal myocardial infarction, sudden cardiac death, development of and death from congestive heart failure [78]. Determining the HS-Omega-3 Index in addition to assessing conventional risk factors provides incremental information, and individuals at medium risk for cardiovascular events can be reclassified into the high or the low risk category. This can be then the basis of a therapeutic consequence. Increasing the HS-Omega-3 Index caused improvements in surrogate and intermediate parameters in pertinent intervention trials (Fig. 3). According to the criteria of the American Heart Association, the HS-Omega-3 Index is a novel biomarker for cardiovascular risk. Among diseases associated with low omega-3 levels are hypertension, congestive heart failure, atrial fibrillation, coronary artery disease, and peripheral atherosclerosis [78].

In contrast, a series of meta-analyses on large intervention trials with EPA and DHA did not find reductions in mortality or cardiovascular events. In the pertinent trials, EPA and DHA were frequently given as capsules at breakfast, thus inadvertently minimizing bioavailability. Moreover, participants were recruited irrespective of baseline levels of EPA and DHA, and the large inter-individual variability in uptake of EPA and DHA was unknown. As demonstrated in one large trial, this design leads to overlapping levels of EPA and DHA throughout the study [79]. Low bioavailability and overlapping levels are thought to contribute substantially to neutral outcomes of many large trials [78]. This is supported by the fact that trials maximizing bioavailability using fatty fish as a source for EPA and DHA, or trials in patients with low baseline levels (e.g., in patients with congestive heart failure) or trials using high doses had positive results [80–82]. However, to convince cardiologists, a new generation of HS-Omega-3 Index-based large intervention study in the cardiovascular area is probably needed.

A low HS-Omega-3 Index or low levels of EPA and DHA in other fatty acid compartments can also be found in issues of brain health: Poor brain development (structure and function), attention-deficit-hyperkinetic disorder (ADHD), poor social behavior, emotional lability, major depression, suicide or cognitive decline (memory, reaction time, executive function) and others [83–85]. Trials using higher doses of EPA and DHA (e.g. >750 mg DHA/day) usually had positive results in these issues of brain function, as substantiated by a series of pertinent meta-analyses [86]. Of note, brain structure and function was found to be improved in healthy individuals.

Furthermore, a low HS-Omega-3 Index or low levels of EPA + DHA are associated with an increased risk for a number of other conditions like stroke [87], osteoporosis [88], rheumatoid arthritis [89], psychopathology and aggression [90], or delayed onset muscle soreness [91]. Since sources of EPA and DHA disappear from our diet and endogenous synthesis is impossible, on a population level symptoms of a deficit become more frequent. When studied, e.g., by national statistic administrations, >80% of the populations had a HS-Omega-3 Index below the target range of 8–11% [92]. The dogma that for healthy people, eating a normal diet, food supplements are unnecessary is wrong in this case [93]. The health of heart, brain and some other organs depends on a sufficient status in EPA and DHA. The widespread deficit in EPA + DHA can thus be conveniently diagnosed and safely treated.

It is well-established, that lipid-lowering therapy with statins can significantly reduce the incidence of cardiovascular disease and the risk of coronary events. However, aggressive lowering of LDL cholesterol by statins comes with the risk of lowering polyunsaturated fatty acid (PUFA) concentrations as well [94], and mitigating the health benefits of omega-3 fatty acid supplementation [95]. Considering the widespread use of this drug group and the inadequate omega-3 PUFA status of many people worldwide [96], it is possible that statin use may be one contributing factor to that situation. The residual risk of cardiovascular events after treatment with statins might be explained in part by the low n-3 PUFA status of patients [97]. Furthermore, a clinically significant additive effect for most cardiovascular events was observed for n-3 PUFAs in patients on statin treatment [98]. Even though statins are established as one of the leading treatment forms to reduce CVD risk, it appears that there is an opportunity to further reduce this risk by omega-3 PUFA.

Based on mechanistic data, and some case reports, concerns have been raised that high doses of omega-3 fatty acids predispose to bleeding events [99, 100]. These concerns, however, could not be substantiated by clinical data:

- in all trials with omega-3 fatty acids in cardiovascular patients (with a total of >75,000 participants), most participants were using some kind of platelet inhibitor like aspirin [78, 101]. Although a safety issue included in the study protocols, increased bleeding was not reported from the omega-3 groups [78, 101].
- a total of 826 patients on phenprocoumon because of a thromboembolic event aged ≥65 years were observed for 3 years. Patients in the highest tertile of their Omega-3 Index lived longer than patients in the lower tertiles, but bleeding events were evenly distributed among tertiles [102].
- a total of 1523 patients with an acute myocardial infarction were treated with platelet inhibitors, anticoagulants and a number of other agents, as demanded by current guidelines. Bleeding events had no relation to the Omega-3 Index of these patients [103].
- intravenous omega-3 fatty acids given before an operation rather improved outcome, but had no effect on bleeding [104, 105].
- the US-American Federal Drug Administration regards up to 3 g/day of EPA plus DHA as safe (Docket No. 91N-0103), while its European counterpart considers up to 5 g/day of EPA as safe (http://www.efsa.europa.eu/en/press/news/120727.htm).

Taken together, although a clinically relevant interaction between drugs affecting platelet function and/or the coagulation system and the nutrient omega-3 fatty acids has been claimed, systematic data and regulatory authorities do not support this claim. Thus, this interaction remains to be proven.

## Drugs and disturbance of microbiota

It is estimated that in the human body, there are approximately the same number of bacterial cells as human cells. The human gut microbiome is a complex ecosystem that is considered to be both an immune and a metabolic organ. Genes, lifestyle, medication, nutrients and their metabolites all influence gut microbiota, however, the effect of this interaction on disease development is uncertain. Nevertheless, several bacteria have been associated with many diseases [106–109] and fecal transplantation is already an emerging method to treat certain conditions.

**Fig. 4:**
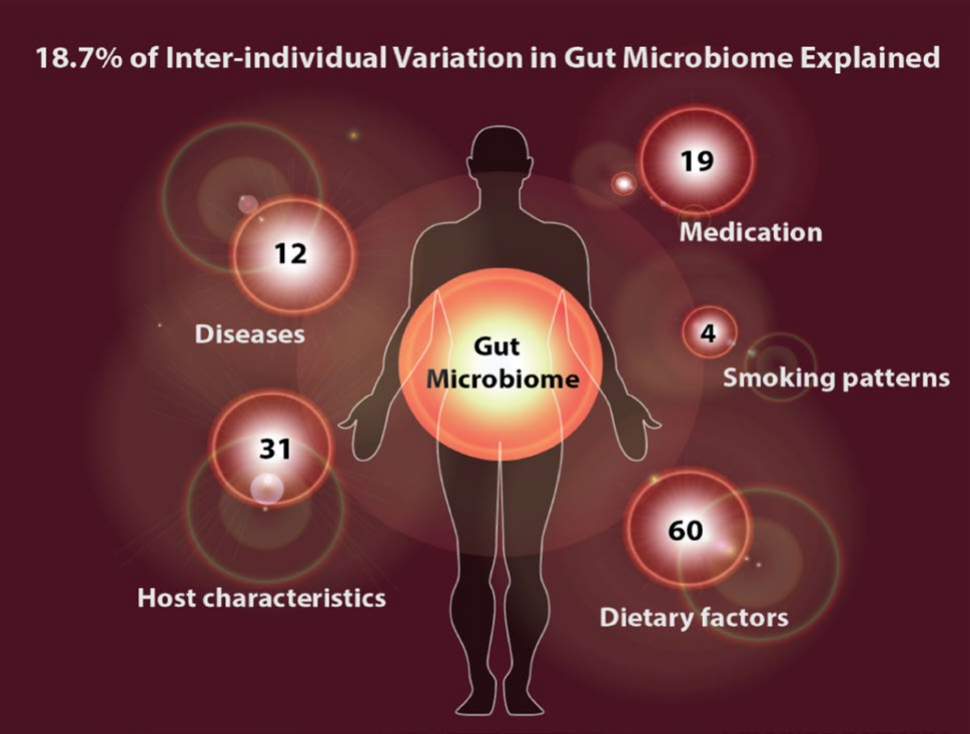
Explained variance of microbiome composition by different factors

In the recent years, population cohorts of healthy individuals have been applied for the analysis of multiple factors influencing microbiota composition. The LifeLines observational follow-up study encompasses in total 165,000 individuals within a 3-generation design. Data collection is complete for multiple questionnaires, different biological, and phenotype information. A follow-up questionnaire is filled out every year, completed with screening every 5 years for a total duration of 30 years [110, 111]. The LifeLines-DEEP project collects extra “omics” data in a subset of the cohort (*n* = 1500), e.g., genome-wide polymorphisms and different metabolites, and analyses the relation between various internal and environmental factors and the microbiome. Within this project, it has been shown that due to the differences in these factors, microbiome composition is highly variable across individuals [112] (Fig. 4).

Further analyses revealed that drugs like proton pump inhibitors (PPI), statins and antibiotics considerably affect gut microbiome composition. PPIs are widely used to treat heartburn symptoms, it is estimated that about 25% of the population takes medication from this drug group when necessary [113]. According to Dutch data, 7% of the population in The Netherlands used omeprazole in 2013 [114]. PPI use is especially overrepresented in obese persons, elderly people, patients with non-alcoholic fatty liver disease or non-alcoholic steatohepatitis (NAFLD, NASH), irritable bowel syndrome (IBS) and inflammatory bowel disease (IBD). A meta-analysis of 300,000 patients showed that PPI use was associated with 65% increase in *Clostridium difficile*-associated diarrhea, and PPIs increased the risk of *C. difficile* recurrence. According to another meta-analysis of 11,280 patients, PPIs increased the risk of *Salmonella*, *Campylobacter* ant other enteric infections [115–118]. Furthermore, PPI use is associated with profound alterations in the gut microbiome, namely a lower diversity can be observed in PPI users. Since PPIs can have negative effect on microbiota composition, it has been proposed that microbiome research in humans should be corrected for PPI use.

A strong correlation of antibiotics has been described with several species of the microbiome. Among others, antibiotics decrease occurrence of *Bifidobacterium longum, Bifidobacterium adolescentes, Roseburia inulinivorans* and increase occurrence of *Lachnospiraceae bacterium 2_1_58FAA* and *Veillonella parvula.* However, in Dutch population the effect of antibiotics on the population level is less strong, than the effect of PPI: the reason for that is low consumption of antibiotics in the Netherlands compared to other countries. Regarding the correlation of statin use with microbiome composition, research is emerging (e.g., *Dorea longicatena* is negatively associated with statin use), with many questions to be answered. This effect can partly be explained by the association of microbiome with lipid levels that have been identified in several human and mouse studies [119].

Currently, what we know is that several drug groups can have a strong effect on gut microbiome. As discussed above, PPIs are not benign in this respect, since they can cause unhealthy changes in the microbiome. Antibiotics might have a small effect on population scale, but this can be substantial for the individuals. Further research faces challenges in dissecting the complex effect of diet, disease, and medication.

## Clinical assessment of interactions of drugs with nutrients: how to approach

As noted before, elderly people are vulnerable in many aspects, including nutritional status. This warrants a systematic assessment of nutrient–drug interactions, starting with classes of drugs that are used in the common complex disorders that account for the global burden of disease of our time. Today, medicine is clearly pharma-dominated where nutrition plays only a confounder role. However, especially lifestyle-related disorders would need a better integrated approach of food and pharma, even for conditions where lifestyle management is already incorporated in clinical guidelines, such as hypertension and diabetes. Clinical trials currently assess adverse effects only, nutritional background is not specified. There is no search for the best nutritional background that fits the study purpose, even for conditions where evidence for interaction of nutritional factors with pharmacological efficacy, or adverse drug effects, was available early in the course of drug development, as was the case for interaction of renin–angiotensin–aldosterone-blockade and sodium intake [120]. Accordingly, investigator brochures focus on toxicity, without assessment of favorable or adverse nutritional conditions that blunt or potentiate drug effect. Post-marketing surveillance and guidelines also address adverse effects; nutritional recommendations are alongside but not integrated with pharmacological recommendations. Moreover, quality indicators focus on drug prescription only. Taken together, neglect of nutritional factors can result in lack of efficacy of pharmacotherapy, even when the latter is considered standard of care, based on evidence from randomized clinical trials, and adopted in current guidelines [121]. Proper consideration of nutritional status in drug action, and assessment of adverse drug effects on nutritional status clearly requires a paradigm change towards inclusion of nutritional assessment throughout the stages of drug development and evaluation, ranging from early stage experimental work, through clinical investigations, approval and eventually post-marketing surveillance. Spontaneous reporting of adverse drug effects in clinical practice should be stimulated, with special attention for nutrition-related events. Strategies for individual patients should include development of drug review protocols, and assessment and integration of nutritional factors and consequences. If possible, clinicians should standardly screen for malnutrition, register measurements of global nutrition status and of dietary supplements used. The proposed framework for categorizing DNIs can be of help structuring these consequences. A necessary condition for working with this framework is more nutritional knowledge amongst health care practitioners and researchers, which might be achieved by integrating nutritional education in the curricula of schools of medicine and pharmacology. Development of a toolbox of nutritional readouts would greatly facilitate this process. One should start with “usual suspects” as models for a systematic approach and use “low hanging fruits” (e.g., quantified assessment of diet) for concept development and relatively rapid benefit. In addition, 24-h urine analysis as a tool for nutritional profiling would allow assessments that are more sophisticated. Examples from several disciplines (e.g., oncology and nephrology) have demonstrated the huge potential gains of proper integration of nutritional and pharmacological management, therefore, engagement of both pharma and food industry in food–drug interactions is of paramount importance for further development. Finally, communicating the outcomes of these recommendations is of crucial interest to integrate DNIs in health care.

## Conclusions

Since from public health perspective the field of drug–nutrition interactions is currently highly undervalued and overlooked, this paper addressed various aspects of this complex topic. We still observe a continuous progress in increasing life expectancy, however, during the last two decades several considerable changes occurred. One example is that in the US, the life expectancy of Caucasians is decreasing, most probably due to lifestyle-related factors like increased stress, drugs abuse, lower socioeconomic status, unhealthy diet and increased prevalence of obesity, leading to the current diabetes epidemic, NAFLD, etc. To reverse this trend and achieve a positive change and improvement in public health, multiple conditions are simultaneously required, most importantly collaboration among all relevant stakeholders and inevitably the governmental will to make improvements happen. Multimorbidity among adults is rapidly escalating with age, in the elderly it is the common status, not an exception any more. This consequently leads to polypharmacy, increasing the risk for major drug–drug interactions. Vitamins as essential nutrients have central functions in metabolism, thus interactions and insufficient availability of vitamins can result in critical impairments of metabolism. Although multiple examples of interactions of drugs with micronutrients are reported, only a small part has been addressed in this paper. These are relatively well-studied cases, but multi-drug interactions have not been tested so far. The question is what can be done in this respect to the benefit of the patients. Since food–pharma–health are interconnected domains, one of the most important actions is to generate higher awareness in the medical community of the potential interactions, e.g., by integrating this topic into the education plans of medical students. On the other hand, nutritional sciences and food industry should also develop an open mind towards interaction and collaboration with the medical domains. It is of paramount importance to further advocate for the integration of drug–nutrition evaluation processes as an essential part of the drug research and development. Strategies for the individual patients should also be developed, by installing drug review protocols, screening for malnutrition and integrating this topic in the general medical advice.
